# Complete Genome Sequence of *Salmonella enterica* Serovar Typhimurium Strain SO2 (Sequence Type 302) Isolated from an Asymptomatic Child in Mexico

**DOI:** 10.1128/genomeA.00253-16

**Published:** 2016-04-14

**Authors:** Claudia Silva, Edmundo Calva, José L. Puente, Mussaret B. Zaidi, Pablo Vinuesa

**Affiliations:** a Departamento de Microbiología Molecular, Instituto de Biotecnología, Universidad Nacional Autónoma de México, Cuernavaca, Morelos, Mexico; b Microbiology Research Laboratory, Hospital General O’Horan and Infectious Diseases Research Unit, Hospital Regional de Alta Especialidad de la Península de Yucatán, Mérida, Yucatán, Mexico; c Programa de Ingeniería Genómica, Centro de Ciencias Genómicas, Universidad Nacional Autónoma de México, Cuernavaca, Morelos, Mexico

## Abstract

The complete genome sequence of *Salmonella enterica* serovar Typhimurium strain SO2, isolated from an asymptomatic child in Mexico, was determined using PacBio single-molecule real-time technology. Strain SO2 has six complete chromosomal prophages, namely, ST104, Gifsy-2, ST64B, Gifsy-1, ELPhiS, and FSL SP-004, and carries a *Salmonella* virulence plasmid.

## GENOME ANNOUNCEMENT

Here, we present the complete genome of *Salmonella enterica* subspecies *enterica* serovar Typhimurium strain SO2, which was isolated in 2002 in Sonora, Mexico, from a stool-culture from an asymptomatic 5-year-old child and reported as strain SOHS 02-20 in an epidemiological surveillance program (1, 2). This strain belongs to multilocus sequence type 302 (ST302), a genotype first described in Mexico for four strains isolated from humans, and later reported for two African strains (http://mlst.warwick.ac.uk/mlst/dbs/Senterica). The ST302 genotype is a derived single-locus variant of the predominant *S*. Typhimurium strain ST19 (2, 3), but is also closely related to ST313, reported for human-invasive strains from Africa (4).

Genomic DNA was extracted by standard protocols (5) and sheared into ~10- to 20-kb fragments for PacBio library preparation and P6-C4 sequencing on one SMRT cell. The continuous long reads were assembled using the HGAP/Quiver-protocol in SMRT portal version 2.3.0.140936.p4 (6), yielding an assembly with two contigs. These were circularized by trimming the terminal repeats with Minimus2 (7), and subjected to three consecutive rounds of read remapping with the RS_Resequencing.1 module for sequence polishing, resulting in a final assembly with a mean coverage of ~175×. The size of the assembled genome is 5,010,007 bp, with a G+C content of 52%, comprising a 4.9-Mb chromosome and a 94-kb serovar-specific *Salmonella* virulence plasmid (pSTV).

Gene calling and annotation were performed with a modified version of Prokka (8). A total of 4,996 genes, including 4,690 coding sequences, and 19 pseudogenes were identified. Additionally, genes for 87 tRNAs, 22 rRNAs, and 1 tmRNA were annotated, plus 165 ncRNAs, 3 CRISPR arrays, 4 riboswitches, and 442 signal peptides. The annotation was manually curated, adding prophage predictions made by the PHAST server (9), and genomic islands detected by IslandViewer3 (10).

Several phage remnants and six complete prophages were located on the chromosome: the frequently found ST104, Gifsy-2, ST64B, and Gifsy-1 phages, and two recently described P2-like phages, ELPhiS and FSL SP-004, originally reported for *S. enteritidis* strains (11) and for a Newport strain (12), respectively. The phage repertoire and the pSTV sequence of strain SO2 were almost identical to those found in the genome of the other Mexican ST302 Typhimurium strain SO3 (13), which was isolated from a human-invasive infection.

### Nucleotide sequence accession numbers.

The complete sequences of the chromosome and the pSTV of *S*. Typhimurium strain SO2 are available in GenBank under accession numbers CP014356 and CP014357, respectively.
